# Oncogene *PRR14* promotes breast cancer through activation of PI3K signal pathway and inhibition of CHEK2 pathway

**DOI:** 10.1038/s41419-020-2640-8

**Published:** 2020-06-15

**Authors:** Xiaolei Ren, Meijun Long, Zhihong Li, Boda Wu, Tao Jin, Chao Tu, Lin Qi, Mei Yang

**Affiliations:** 1 Department of Orthopedics, The Second Xiangya Hospital, Central South University, Changsha, China; 2 Hunan Key Laboratory of Tumor Models and Individualized Medicine, The Second Xiangya Hospital, Central South University, Changsha, China; 3 Breast Cancer Center, The Third Affiliated Hospital of Sun Yat-sen University, Guangzhou, China; 4 Department of Medical Genetics, The Second Xiangya Hospital, Central South University, Changsha, China; 5 Department of Gastroenterology, The Second Xiangya Hospital, Central South University, Changsha, China; 6 Center for Medical Genetics, School of Life Sciences, Central South University, Changsha, China

**Keywords:** Breast cancer, Checkpoint signalling, Checkpoints

## Abstract

Nuclear envelope component PRR14 has been detected to be upregulated in varieties of cancers, especially in breast cancer. But its role in breast carcinogenesis is poorly understood. In this study, we show PRR14 contributes to breast carcinogenesis mainly through overexpression, which derives from elevated transcription and gene amplification. Increased PRR14 expression promotes breast cancer cell proliferation and tumor formation. Biochemical analysis reveals, in addition to previously reported activation of PI3-kinase/Akt/mTOR pathway, PRR14 overexpression regulates cell cycle in breast cancer by inhibiting CHEK2’s activation, followed with the deregulation of DNA damage pathway. In correspondence, CHEK2 and PRR14 show opposite impact on breast cancer patients receiving chemotherapy. Collectively, our study is the first to document the oncogenetic role of *PRR14* in breast cancer, which protects cells from apoptosis and stimulates proliferation by activating the PI3-kinase/Akt/mTOR pathway and inhibiting the CHEK2 pathway. Both of these pathways are of great influence in breast cancer and PRR14 appears to be their novel interacting node, which renders patients more resistance to chemotherapy and provides a potential therapeutic target in breast cancer.

## Introduction

Morphologically, cancer cells are characterized by alterations of nuclear structure, including abnormal size and shape, irregular numbers and sizes of nucleoli and different chromatin texture^1^. These alterations are so characteristic that changes in cell and tissue structure have remained the gold standard for diagnosis of cancer for decades, and the assessment of a biopsy sample is still required to confirm the diagnosis for most cancers^2^. However, the specific nuclear components involved, the underlying molecular mechanisms and the implications for cancer progression remain elusive, which may be explained by our currently limited understanding of nuclear architecture and their functional relevance. Breast cancer is the most prevalent cancer in women and the second most prevalent cancer overall, in which, nuclear pleomorphism is not only employed to differentiate cancer cells from normal tissue but graded and correlated with clinical aggressiveness and patients’ outcome^3–5^.

Cell nucleus separates genetic information from the rest of the cells by nuclear envelope, which provides structural support to the nucleus and mediates its exchange of materials and signals with cytosol^6^. As a result, the nuclear envelope is involved in most nuclear activities, including the mechanical stability and shape of the nucleus, DNA replication and transcription, chromatin organization, cell-cycle regulation, cell development and differentiation, nuclear anchoring and migration, centrosome positioning, apoptosis and so on^7,8^. Specific defects in nuclear envelope genes lead to aberrant nuclear morphology, for example *Lamin A/C*^9^, *Emerin*^10^, *Sun1*^11^, *TOR1AIP1*^12^, *Nesprin-1* and -*2*^13^ etc. During tumorigenesis, the components of the nuclear envelope undergo significant alterations and several nuclear envelope proteins have been found dysregulated in various cancers^14–17^. Therefore, it can be hypothesized that the nuclear envelope has a key role in connecting nuclear pleomorphism with its functional alteration during tumorigenesis.

Proline rich 14 (PRR14) is a newly identified component of the nuclear envelope^18^. Like many other nuclear envelope proteins, PRR14 is involved in the regulation of nuclear morphology and its deficiency results in nuclear morphological alterations^18,19^. Structurally, PRR14 tethers heterochromatin to the nuclear lamina and mediates their functional interaction, disruption of which almost completely blocks myogenesis^19^. By comparing expression between cancerous tissue and normal tissue in The Cancer Genome Atlas (TCGA), *PRR14* transcripts are detected to be elevated in various types of cancers^20^. Its role in tumorigenesis has been firstly identified in lung cancer, in which, *PRR14* functions as an oncogene by activating the PI3K/AKT/mTOR signaling pathway^21^.

Serine/threonine kinase CHEK2 is implicated in pathways that govern DNA repair and cell-cycle checkpoint regulation. It negatively regulates cell-cycle progression during unperturbed cell cycles^22^. When genotoxic stress is detected, CHEK2 is phosphorylated at Thr68 by ATM, which enables its efficient activation^23,24^, followed by phosphorylation of its substrates, including but not limited to P53^25^, BRCA1^26^, BRCA2^27^, PML^28^, E2F-1^29^, CDC25A^30^ and CDC25C^31^ etc. These phosphorylated substrates block cell-cycle progression at all cell-cycle checkpoints (G1/S, G2/M and spindle assembly checkpoints), prevent entry into mitosis, repair DNA damage and regulate apoptosis. Some of these substrates, such as P53, BRCA1 and BRCA2, are key players in tumorigenesis, especially in breast cancer. In consistence, missense or deleterious mutations in *CHEK2* causing loss of its kinase function, though very rare, have been correlated with different types of cancers, particularly breast cancer. Therefore, tumor suppressor *CHEK2* is widely accepted as a driver gene during tumorigenesis^32,33^.

Although the expression of *PRR14* is upregulated in various types of cancers, the upregulation is most significant in breast cancer^20^. Here, using an integrated approach consisting of bioinformatic analysis, in vitro and in vivo biochemistry studies, we explore *PRR14*’s function in breast cancer. Our observation confirms PRR14’s overexpression and its effect on activating the PI3K/AKT/mTOR signaling pathway in breast cancer. In addition, PRR14 is newly found to regulate cell cycle by inhibiting tumor suppressor CHEK2, which further strengthens its function as an oncogene in breast cancer and confers cancer cell resistance to chemotherapy.

## Results

### PRR14 is amplified and overexpressed in human breast cancer samples

**Fig. 1:**
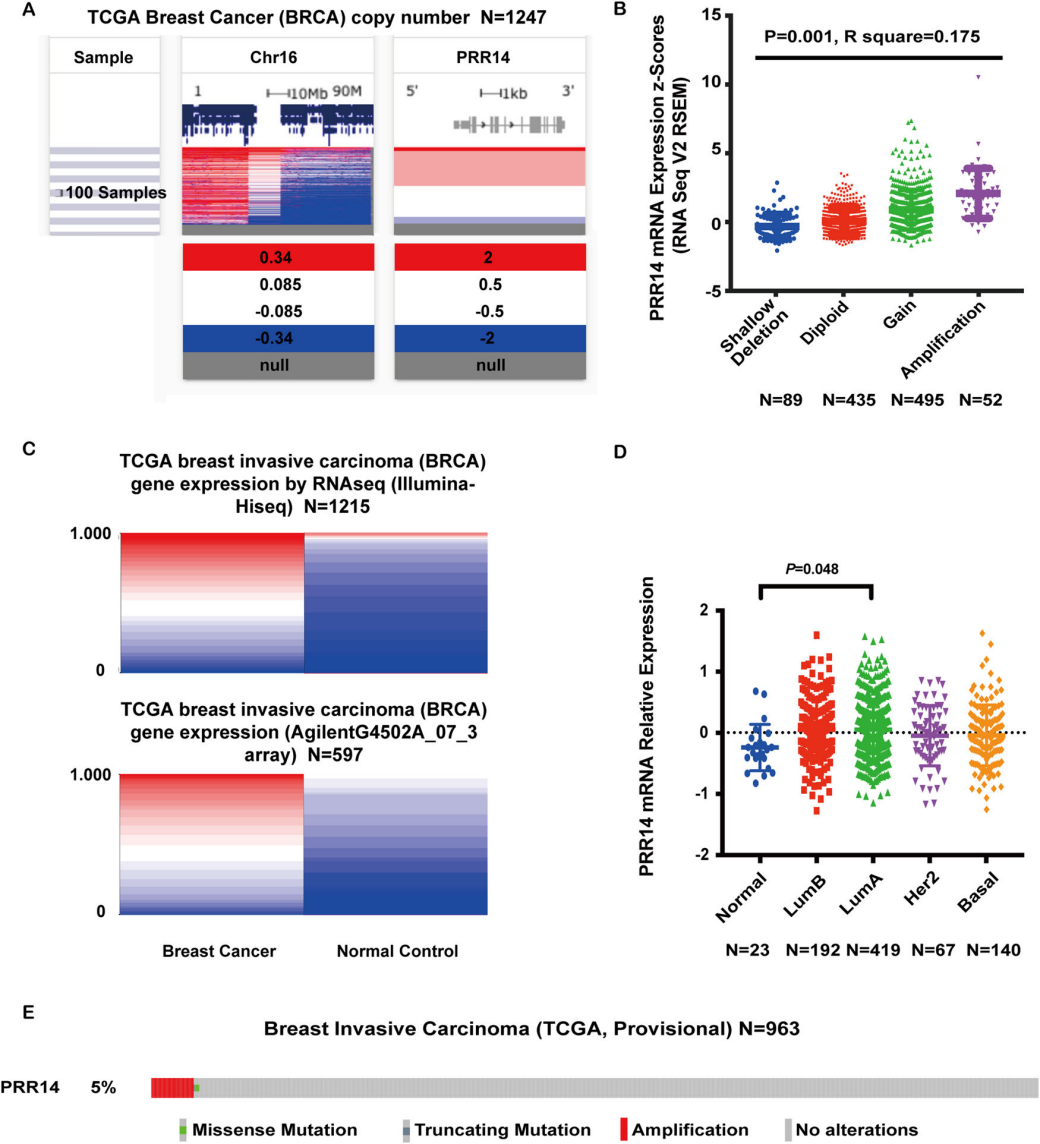
*PRR14* is amplified and overexpressed in breast cancer. **a** Gene copy number information of Chr16 and *PRR14* are taken from UCSC Cancer Browser. **b** The relationship between *PRR14* gene mRNA expression and its copy number variation is analyzed by one-way ANOVA analysis. Mean ± s.d. is presented. **c** *PRR14* expression data in TCGA, detected either by RNAseq or array, are retrieved from UCSC Cancer Browser. The implemented in UCSC Cancer Browser statistical analysis using Student’s *t*-test and adjusting the false discovery rate using the Benjamini–Hochberg procedure identified statistical significance (*P* < 0.05) with higher expression of *PRR14* in cancer samples. **d** *PRR14* expression in breast cancer molecular subtypes: Normal-like (Normal), LumB, LumA, Her2 and Basal, is shown and compared by one-way ANOVA analysis. Mean ± s.d. is presented. **e** The genetic information of *PRR14* in Breast Invasive Carcinoma in TCGA is profiled.

*PRR14* resides in 16p11.2, a region frequently amplified in breast cancer^34^. Data mining from TCGA shows consistent copy number increase in this region (Fig. 1a), which positively correlates with *PRR14* transcription in breast cancer (Fig. 1b, *P* = 0.001, *R*^2^ = 0.175). As expected, the transcription of *PRR14* in breast cancer, measured either by RNAseq or gene expression array, is significantly enhanced (Fig. 1c). In light of the fact that transcriptome in breast cancer differs greatly among subtypes, *PRR14*’s transcription among different intrinsic molecular subtypes defined by PAM50 (Normal-like, Basal, LumA, LumB and Her2) is compared^35–37^. One-way ANOVA analysis demonstrates that the difference is rather small, which only exists between Normal-like subtype and LumA subtype (Fig. 1d, *P* = 0.048). Furthermore, genetic analysis indicates that *PRR14* is rarely mutated and the majority of genetic alterations in breast cancer are gene amplification (Fig. 1e), which imply that gene overexpression instead of pathogenic mutation has an important role in regulating *PRR14*’s function during breast tumorigenesis.

**Fig. 2:**
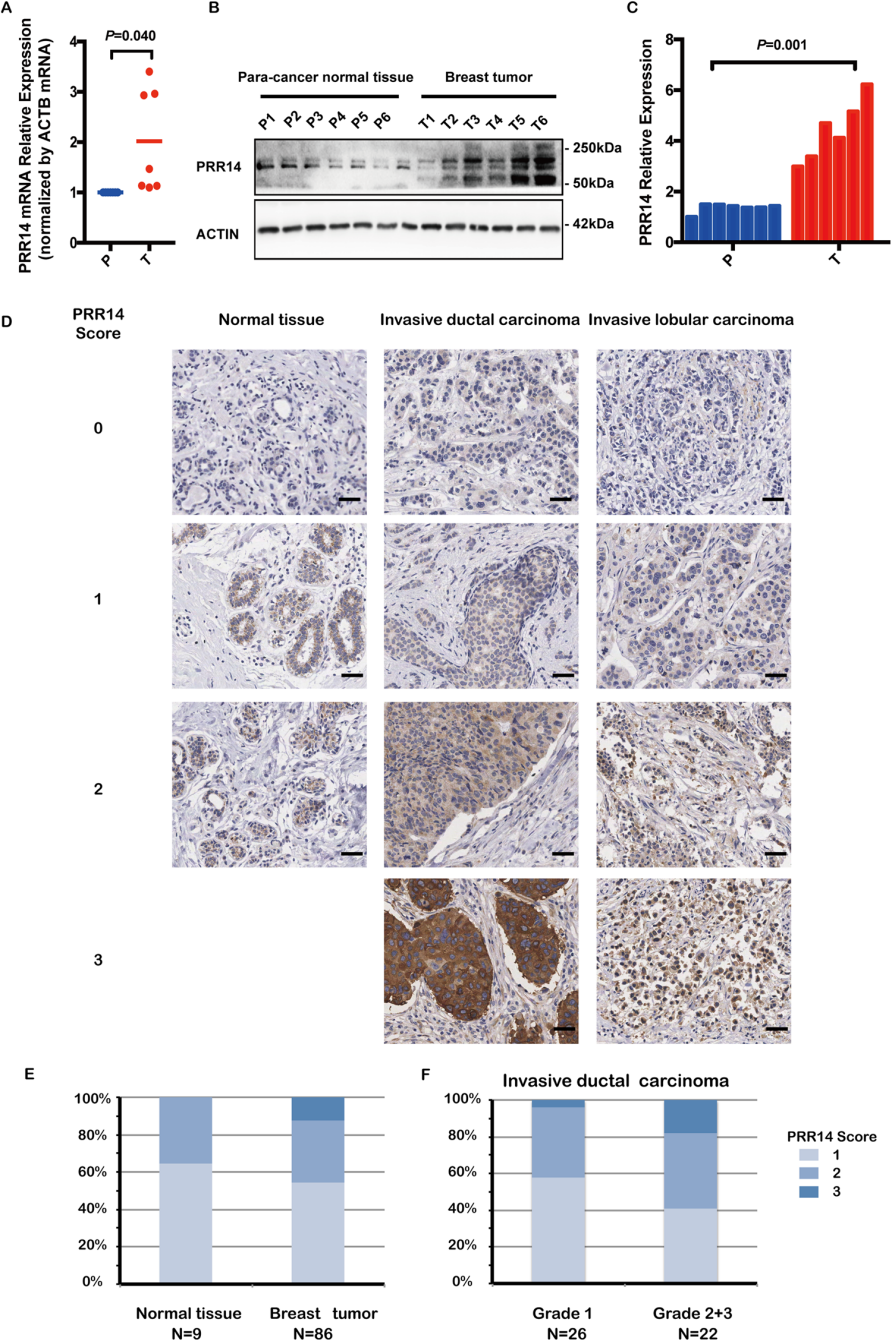
The upregulated expression of PRR14 is confirmed in human breast samples. *PRR14* mRNA (**a**) and protein (**b**) expression in breast cancer and para-carcinoma breast tissue are detected by qRT-PCR and immunostaining, respectively. The protein level is quantified (**c**). Both the transcription and expression are normalized by ACTB and statistically analyzed by paired two-tailed Student’s *t*-test. Representative images of IHC staining for PRR14 with different staining intensity in a human breast cancer tissue microarray are presented (**d**). The data are presented as the percentage of samples with different PRR14 staining intensity (**e**, **f**).

To corroborate the result from bioinformatic analysis, we detect *PRR14*’s expression in human breast tissues. Using qRT-PCR analysis, the upregulated transcription of *PRR14* in breast cancer is confirmed in 7 pairs of matched human breast cancerous and adjacent normal tissues (Fig. 2a, *P* = 0.040). Consistently, the upregulation is also observed in protein level in 6 out of 7 pairs of the tissues (1 pair of tissue is missing for inadequacy) (Fig. 2b, c, *P* = 0.001). Further, we measure PRR14’s expression by immunohistochemical staining of human breast cancer tissue microarray (TMA), which comprises normal breast tissue, invasive ductal carcinoma and invasive lobular carcinoma (Fig. 2d, Supplementary Table 1). About 7% of the samples (16/192) are undetectable and about 42% of the samples (81/192) are considered PRR14 negative (score = 0). In the rest 95 PRR14-positive samples, both the maximal immunoreactivity and the percentage of stronger immunoreactivity of PRR14 in breast tumor are higher than those in normal tissue (Fig. 2e). When compared among graded invasive ductal carcinoma, a higher percentage of stronger immunoreactivity of PRR14 is observed in higher grade (Fig. 2f).

### PRR14 promotes breast cancer tumorigenesis in breast cancer

**Fig. 3:**
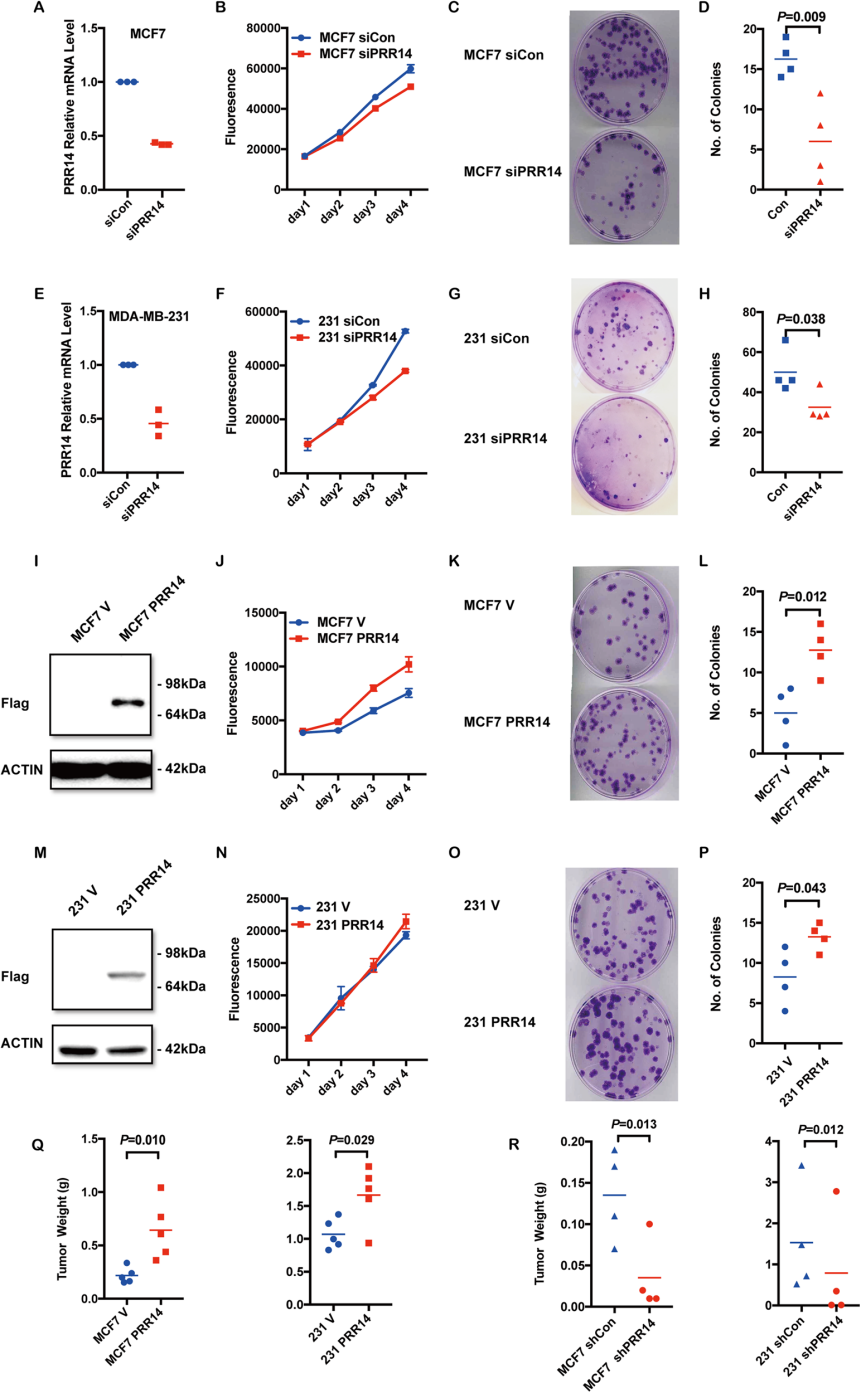
PRR14 promotes breast tumorigenesis. siRNA is transfected into breast cancer cell lines MCF7 and MDA-MB-231. Compared with the control sequence, *PRR14*-specific siRNA decreases its mRNA level to <50% (**a**, **e**). Growth curves following transfection are monitored with the Alamar Blue Assay. Mean ± s.d. is presented (**b**, **f**). And Colony formation is performed at the same time (**c**, **g**). Colony numbers are calculated and statistically analyzed by two-tailed Student’s *t*-test. Mean is presented. (*n* = 3) (**d**, **h**). The overexpression of PRR14 in established MCF7 and MDA-MB-231 cell lines is confirmed by immunostaining (**i**, **m**). Also, growth curve (**j**, **n**) and colony formation assay (**k**, **o**) are performed (*n* = 3) (**l**, **p**). Female nude mice are injected with either PRR14-overexpressing or its control cell lines as well as PRR14-depleted or its control cells in both flanks and palpable tumor formed. Tumors are weighted and paired two-tailed Student’s *t*-test is employed to determine the significance of the difference between the two groups (**q**, **r**).

To determine whether PRR14 has a functional role in breast cancer carcinogenesis, we employ two commonly used breast cancer cell lines, MCF7 and MDA-MB-231. Firstly, a mixture of three different siRNAs targeting three different sites of *PRR14* is used to decrease its expression. qRT-PCR analysis shows that *PRR14*’s transcription is decreased to around 50% (Fig. 3a, e) in both cell lines, which results in lower protein level (Supplementary Fig. A) and slower proliferation (Fig. 3b, f) and lower colony formation capacity (Fig. 3c, d and g, h). In contrast, ectopic expression of PRR14 in retrovirus-mediated gene transfer established stable cell lines, which is confirmed by immunoblot (Fig. 3i, m), leads to a significant increase in both proliferation rate (Fig. 3j, n) and colony formation capacity (Fig. 3k, l and o, p). Altogether, these results indicate that PRR14 promotes breast cancer tumorigenesis.

To confirm *PRR14*’s function as an oncogene in breast cancer in vivo, we perform xenograft experiments. MCF7 and MDA-MB-231 cells stably expressing PRR14 and their matched control cells are subcutaneously implanted into either side of nude mice. In agreement with the in vitro data, enhanced PRR14 in both cell lines results in significantly increased tumor volume as compared to controls (Supplementary Fig. B, Fig. 3q, MCF7: *P* = 0.010; MDA-MB-231: *P* = 0.029). The tumor-promoting activity of PRR14 is validated by a complementary knockdown experiment (Supplementary Fig. C, D, Fig. 3r, MCF7: *P* = 0.013; MDA-MB-231: *P* = 0.012). The result shows that less PRR14 expression derived from short hairpin RNA leads to significantly reduced tumor formation in mice.

### PRR14 activates the Akt/mTOR signaling pathway

**Fig. 4:**
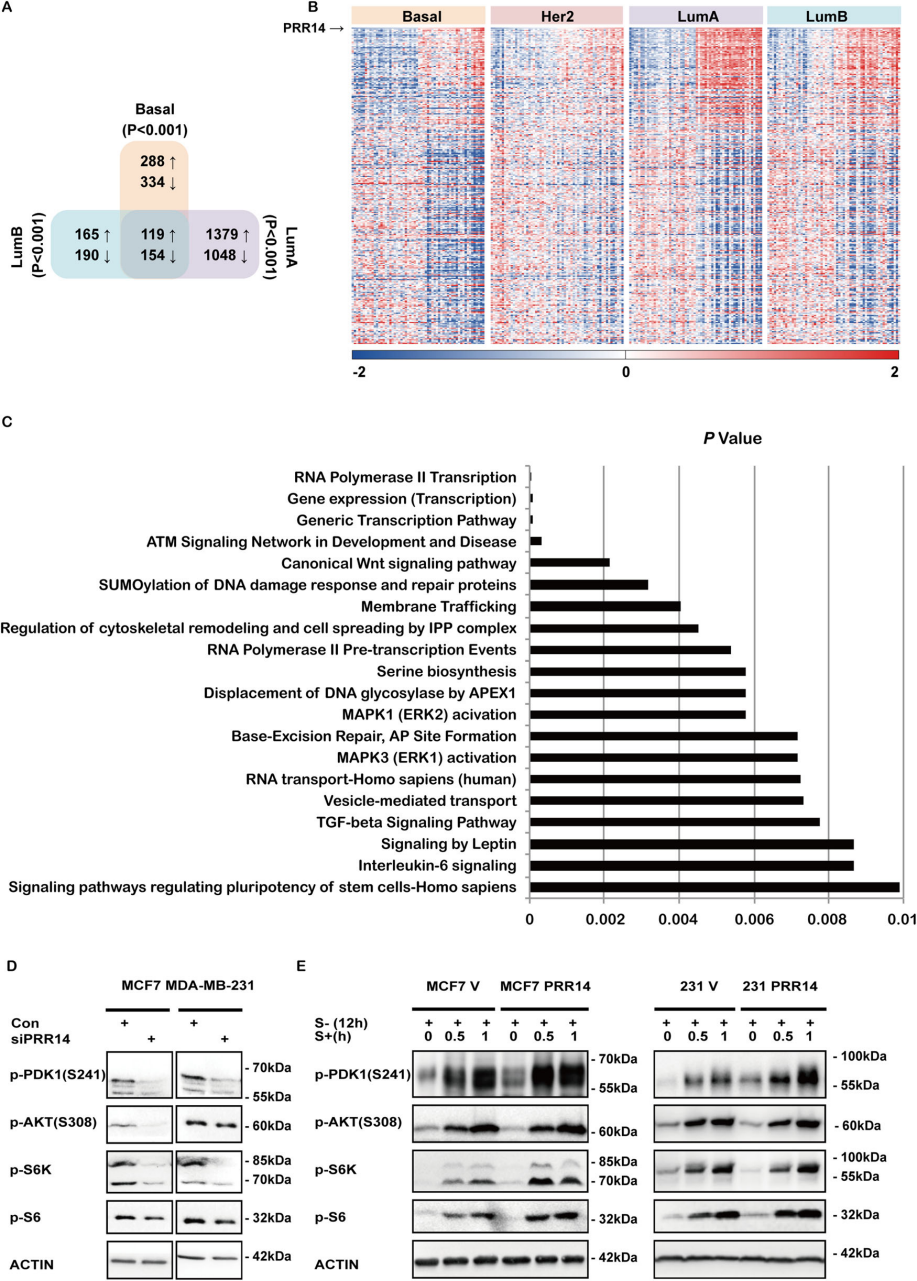
PRR14 activates the PI3K signal pathway. Venn diagram presenting comparative analysis of DE genes between high- and low- PRR14-expressing Basal, LumA and LumB cases deposited in the TCGA database. The cutoff of *P* < 0.001 is considered significant to determinate DE transcripts (**a**). Heatmap presenting 273 common DE genes in Basal, Her2, LumA and LumB subtypes of breast cancer (**b**). The gene expression levels are normalized and log2-transformed, reaching from −2 to +2 that corresponds to blue and red colors on heatmap, respectively. *P-*value and over-represented pathways from 273 common DE genes are analyzed using ConsensusPathDB (**c**). Key components of the PI3K/Akt/mTOR signaling pathway are detected by immunostaining in both MCF7 and MDA-MB-231 cell lines transfected with siRNA to reduce PRR14 expression (**d**), as well as established PRR14-overexpressing and its control cell lines (**e**). Serum is added to the medium for indicated time after 12-h-serum starvation to stimulate the signaling pathway in PRR14-overexpressing and the control cell lines.

To explore the underlying mechanisms of PRR14’s function in breast cancer, we perform differential expression analysis across transcriptome deposited in TCGA BRCA database as previously described^21^. Thirty high-PRR14- and low-PRR14-expression samples are retrieved from each subtype respectively, including Basal (139 cases), LumA (419 cases) and LumB (192 cases) subtypes, and perform differential expression analysis. Normal-like (11 cases) and Her2 (67 cases) subtypes are excluded for insufficient samples. The comparative analysis between high-PRR14- and low-PRR14-expressing samples within the rest 3 subtypes show 273 common differentially expressed (DE) transcripts (*P* < 0.001) including 119 increased and 154 decreased with PRR14 (Fig. 4a) (Supplementary Table 2). Similar expression pattern of the 273 genes is observed in Her2 subtype as well (Fig. 4b). Over-representation analysis of the 273 commonly DE genes indicates 20 pathways are statistically significantly over-represented (Fig. 4c, *P* < 0.01, Supplementary Table 3). The top three hit pathways are RNA Polymerase II Transcription (*P* = 4.78E−05, *q* = 0.0112), Gene expression (Transcription) (*P* = 6.98E−05, *q* = 0.0112) and Generic Transcription Pathway (*P* = 8.47E−05, *q* = 0.0112). All of the three pathways involve the AKT/mTOR signal pathway to regulate global gene expression, which is commonly activated in various types of cancer^38,39^, and its activation by PRR14 has been identified in lung cancer^21^. We assume activation of the signal pathway may be a common mechanism of *PRR14*’s function as oncogene in both lung cancer and breast cancer. To test this assumption, the activity of the signal pathway is checked, which is regulated and reflected by the phosphorylation of PDK1, AKT, S6K and S6. The immunoblot result demonstrates that PRR14 depletion by RNAi reduces the phosphorylation (Fig. 4c), whereas PRR14-overexpressing cells show elevated phosphorylation than their control cells when stimulated by serum after serum starvation for 12 h. This indicates PRR14’s upregulation activates the AKT/mTOR signal pathway and vice versa in breast cancer cells (Fig. 4d).

### PRR14 is able to regulate cell cycle

**Fig. 5:**
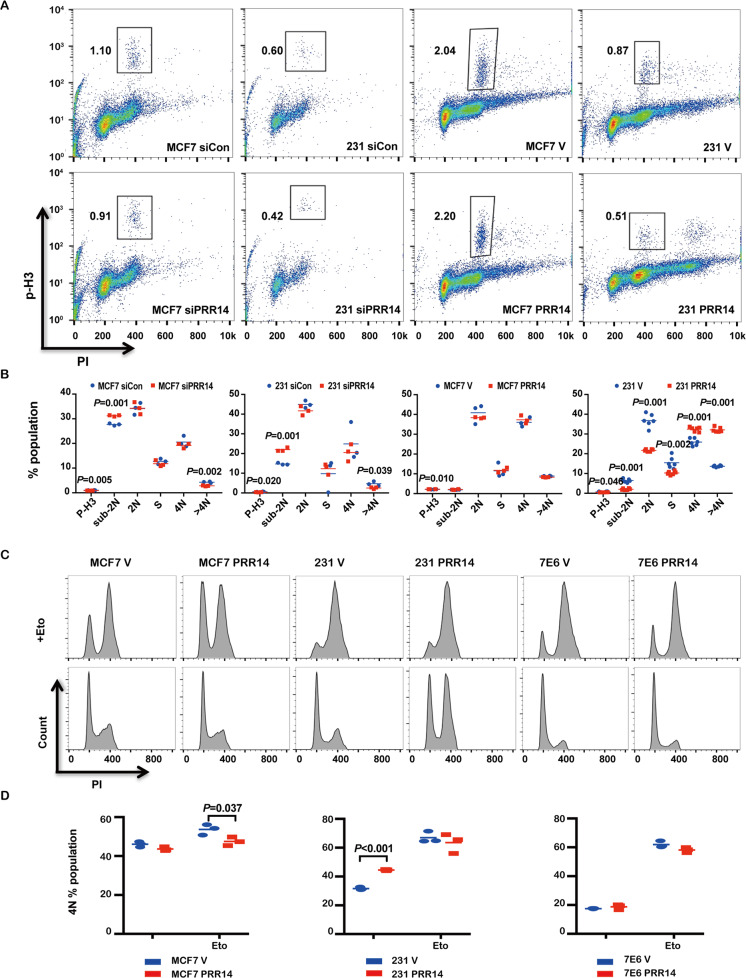
PRR14 regulates cell cycle. MCF7 and MDA-MB-231 cells transfected with PRR14-specific siRNA or negative control as well as established PRR14-overexpressing and its control cell lines (**a**) are stained with PI and p-H3. Cell-cycle profile is obtained by FACS analysis and statistically analyzed by two-tailed Student’s *t*-test (*n* = 3) (**b**). Established PRR14-overexpressing and control cell lines are treated w/wo 5 μg/ml Eto. 24 h later, cells are harvested to stain with PI and cell-cycle profile is obtained by FACS analysis (**c**). The percentage of the 4N fraction is analyzed by two-tailed Student’s *t*-test (*n* = 3) (**d**).

We notice that another critical signal pathway in breast cancer, ATM Signaling Network in Development and Disease (Fig. 4c, *P* = 0.000321, *q* = 0.0318, Supplementary Table 3), is also regulated by PRR14 in the result of the over-representation analysis. As the ATM signaling pathway is closely involved with DNA damage repair and cell-cycle regulation, we check PRR14’s impact on cell cycle. When the expression of PRR14 is depleted by RNAi, both MCF7 and MDA-MB-231 cells analyzed by FACS analysis show obvious increase in cell death, indexed by sub-2N fraction, slightly yet still significant decrease in proliferation, reflected by p-H3, and significant reduction in heteroploidy, indexed by >4N fraction. Whereas in stable cell lines, the influence of PRR14 overexpression is not the same between MCF7 and MDA-MB-231 cells. Compared with the control cells, PRR14-overexpressing MCF7 cells demonstrate a slight increase of p-H3-positive fraction (*P* = 0.010). While in MDA-MB-231 cells, stable overexpression of PRR14 results in decreased p-H3-positive fraction (*P* = 0.046), sub-2N fraction (*P* = 0.001), 2N fraction (*P* = 0.001) and S fraction (*P* = 0.002) as well as increased 4N fraction (*P* = 0.001) and >4N fraction (*P* = 0.001) (Fig. 5a, b).

When cells are treated with Eto to induce DNA damage, P53 is activated by CHEK2 and stimulates *P21* transcription as a transcription factor, which finally blocks cell cycle in G2-phase^40^, indexed as 4N in FACS analysis (Fig. 5c, d). In MCF7 cells, the signal pathway is functional and the population of cells arrested in G2-phase is significantly less in PRR14-overexpressing cells as a reflection of reduced activity of CHEK2 (*P* = 0.037). In P53 mutant MDA-MB-231 cell lines, relevant CHEK2 signal pathway is not functional and cell cycle is arrested through other pathways^41^. Consequently, the percentage of arrested G2-phase cells does not show obvious difference between PRR14-overexpressing cell line and its control cell line. Further, when we block P53’s activity in MCF7 cells by HPV-16 E6 (7E6)^42,43^ (Supplementary Figs. E, F), the difference in the percentage of arrested G2-phase cells disappears.

### PRR14 inhibits CHEK2

To elucidate the underlying mechanism of cell-cycle regulation by PRR14, we further compare the protein expression between *PRR14* genetically altered (amplified + mutated) and genetically unaltered samples in the TCGA database (Supplementary Table 4), considering the ATM signaling pathway is mainly regulated through phosphorylation. A total of 30 proteins are significantly differentially expressed (*P* < 0.01, *q* < 0.05), among which PDK1 and its activated form p-PDK1 (S241)^44^ are the two most increased proteins in the presence of *PRR14* genetic alterations. This is consistent with the previous result that PRR14 activates the Akt/mTOR signaling pathway in breast cancer. Besides, several cell-cycle-related proteins appeared in the list, including CDK1, p-CDKN1B (T198), RAD50, RAD51 and p-CHEK2 (T68). Combining the results of proteins and transcripts regulated by PRR14 overexpression and its multifunction in cell-cycle regulation, we focus our research on *CHEK2*.

**Fig. 6:**
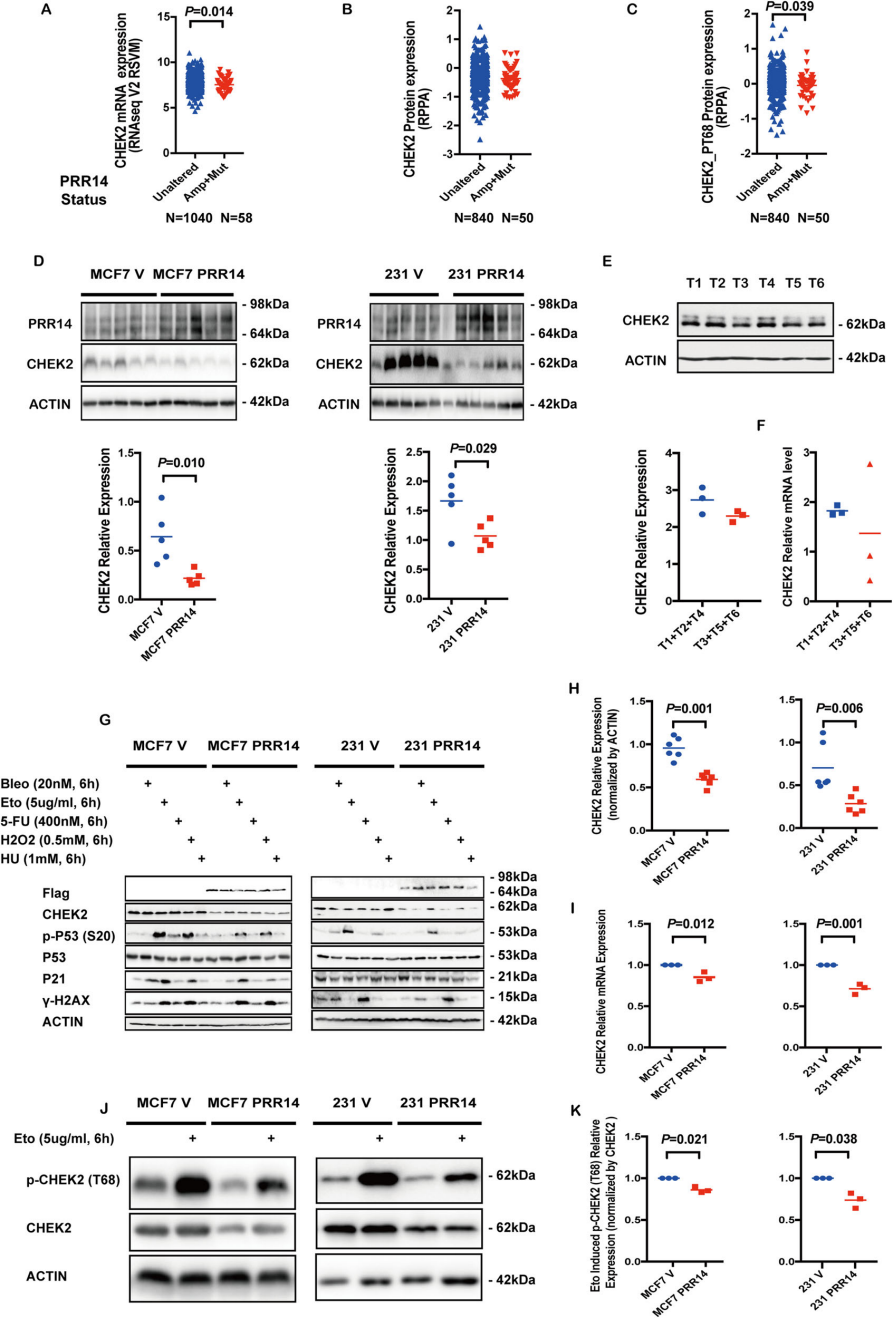
PRR14 inhibits CHEK2. CHEK2 mRNA expression (**a**), CHEK2 protein expression (**b**) and p-CHEK2 (T68) protein expression (**c**) in PRR14 genetically unaltered and altered (amplified and mutated) breast cancer cases in TCGA database are statistically analyzed by two-tailed Student’s *t*-test. CHEK2 protein expression is detected by immunostaining in xenografts in nude mice from established MCF7 cell lines and MDA-MB-231 cell lines (**d**), as well as human breast cancer (**e**). CHEK2 transcription in human breast cancer is also detected by qRT-PCR (**f**). The data are quantified and two-tailed Student’s *t*-test is employed to determine the significance of the difference. Established MCF7 and MDA-MB-231 (**g**) PRR14-overexpressing and control cell lines are treated with various of genotoxic chemicals including Bleo, Eto, 5-FU, H2O2 and HU at indicated concentrations for indicated time. Key components of the ATM/CHEK2/P53 signaling pathway are detected by immunostaining. And CHEK2 protein expression (**h**) and mRNA expression (**i**) are detected by immunostaining and qRT-PCR, respectively. The data are analyzed by two-tailed Student’s *t*-test. Established MCF7 and MDA-MB-231 (**j**) PRR14-overexpressing and control cell lines are treated with Eto at indicated concentration for indicated time to induce p-CHEK2 (T68), which is detected by immunostaining and quantified and normalized by CHEK2 total protein (**k**). The data are analyzed by two-tailed Student’s *t*-test.

Comparison between *PRR14* genetically altered and unaltered breast cancer samples shows that *CHEK2* transcription is reduced (Fig. 6a, *P* = 0.014) in *PRR14* genetically altered breast cancer samples, accompanied by not significantly less protein (Fig. 6b) and significantly less activated form p-CHEK2 (T68) (Fig. 6c, *P* = 0.038). We check the expression of CHEK2 and PRR14 in the tumors from our previous xenograft experiment (Fig. 6d) and human breast cancer samples (Fig. 6e). Xenografts derived from PRR14-overexpressing cell lines show higher PRR14 protein and lower CHEK2 protein level than xenografts derived from the control cell lines (Fig. 6d, MCF7 V vs. MCF7 PRR14, *P* = 0.010; 231 V vs. 231 PRR14, *P* = 0.029). Human breast cancer samples are classified into two groups according to PRR14’s protein level. Both the protein level and mRNA level of CHEK2 are lower in the high-PRR14 group (T3 + T5 + T6, Fig. 6e, f), but the difference is not significant. The activity of CHEK2 signal pathway is also checked in cell lines (Fig. 6g). When cells are treated with genotoxic chemicals, including Bleo, Eto, 5-FU, H_2_O_2_ and HU, the ATM-CHEK2 signal pathway is activated. Compared with control cell lines, both PRR14-overexpressing cell lines demonstrate similar level of total P53 protein, but reduced level of p-P53 (S20), indicating less activity of CHEK2, whereas DNA damage marker, γ-H2AX, another product of ATM activation^45^, shows little difference. This excludes the possibility that ATM contributes to CHEK2’s reduced activity in PRR14-overexpressing cells. In MCF7 cells with wild-type *P53*, the cell-cycle inhibitor P21, transcriptionally induced by p-P53, is less in the presence of DNA damage as a reflection of less p-P53 (Fig. 6g). Whereas in mutant *P53* expressing MDA-MB-231 cells, the transcription of P21 cannot be induced by P53, even though it is phosphorylated and activated, and P21 remains constant (Fig. 6g). This implies the effect of PRR14’s overexpression differs in cells with wild-type and mutant *P53*.

Having shown that the activity of CHEK2 is inhibited by PRR14, we seek to understand the underlying mechanism. Both CHEK2 protein level (Fig. 6g, h, MCF7 V vs. MCF7 PRR14, *P* = 0.001; 231 V vs. 231 PRR14, *P* = 0.006) and mRNA (Fig. 6i, MCF7 V vs. MCF7 PRR14, *P* = 0.012; 231 V vs. 231 PRR14, *P* = 0.001) level is lower in PRR14-overexpressing cell lines (Fig. 6g–i, MCF7 V vs. MCF7 PRR14, *P* = 0.001; 231 V vs. 231 PRR14, *P* = 0.006). As expected, p-CHEK2 (T68) is less in PRR14-overexpressing cells than that in control cells (Fig. 6j, k). Furthermore, when we normalize the level of CHEK2’s activation by its total protein level instead of the internal control β-ACTIN protein to eliminate the influence of different CHEK2 protein levels between cell lines, p-CHEK2 (T68) is still less in cells with elevated PRR14 (Fig. 6k, MCF7 V vs. MCF7 PRR14, *P* = 0.021; 231 V vs. 231 PRR14, *P* = 0.038).

### PRR14 regulates cell cycle through CHEK2

**Fig. 7:**
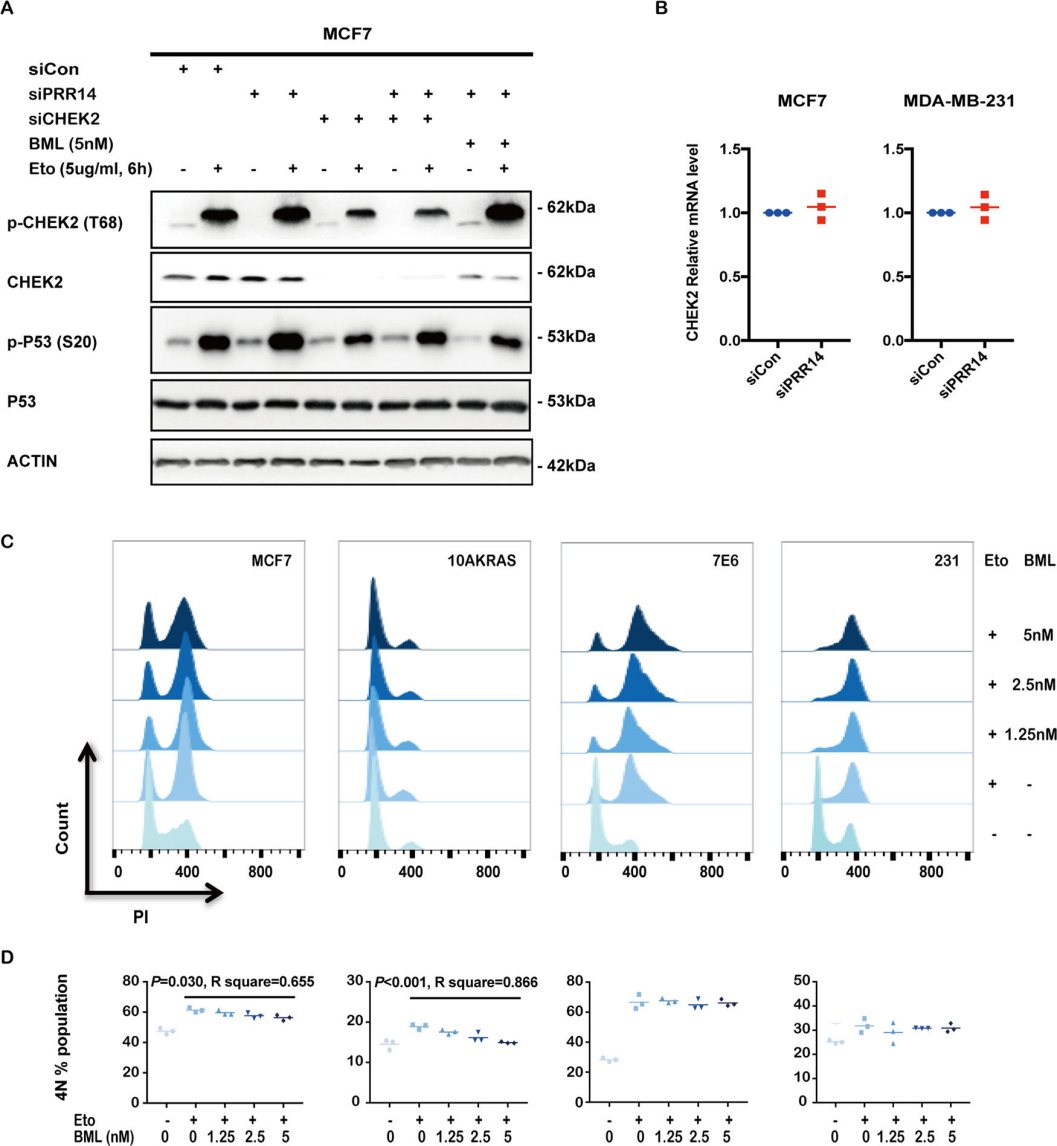
CHEK2 mediates PRR14’s regulation on breast cancer cell cycle. MCF7 cells transfected with indicated siRNA sequences for 48 h are treated w/wo 5 μg/ml Eto for 6 h, then cells are harvested for immunostaining (**a**). PRR14 in cells is depleted by RNAi transfection for 48 h, and CHEK2 mRNA level are quantified by qRT-PCR in both MCF7 and MDA-MB-231 cell lines (**b**). MCF7, 10AKRAS, 7E6 and MDA-MB-231 cell lines are treated with increasing concentration of CHEK2 inhibitor BML for 24 h and followed with a 24 h treatment of Eto at 5 μg/ml. Cells are harvested to stain with PI followed with FACS analysis (**c**). The percentage of the 4N fraction is analyzed by one-way ANOVA analysis (*n* = 3) (**d**).

Then, we check the functional consequences of CHEK2’s inhibition by PRR14. In MCF7 cell line, PRR14 depletion by RNAi increases p-CHEK2 (T68) in the presence of Eto but shows no effect on CHEK2 total protein. Consistently, enhanced p-P53 (S20), direct product of CHEK2 activity, is detected. CHEK2 depletion by RNAi greatly decreases its protein as well as its active form p-CHEK2 (T68). CHEK2-specific inhibitor BML inhibits CHEK2 activity, but shows no effect on both its protein and p-CHEK2 (T68) level. Accordingly, p-P53 (S20) is reduced by these two treatments, even in the presence of PRR14 depletion (Fig. 7a). When we check CHEK2’s transcription in response to PRR14 depletion, no significant difference is observed, which is consistent with its protein level (Fig. 7b).

**Fig. 8:**
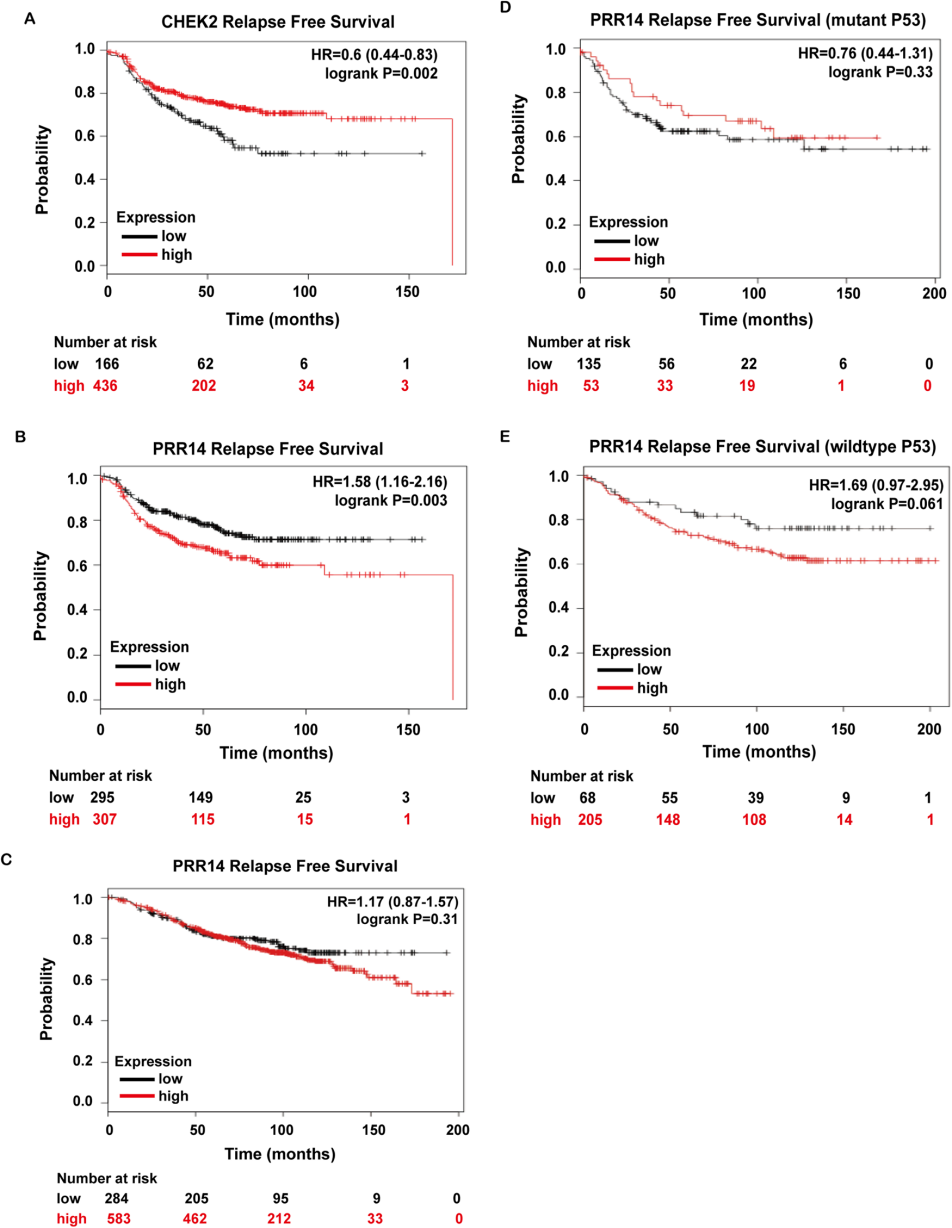
PRR14’s effect on the survival of breast cancer patients. KM survival curves of breast cancer patients receiving chemotherapy are stratified by their expression levels of either CHEK2 (**a**) or PRR14 (**b**). For comparison, KM survival curves of breast cancer patients receiving endocrine therapy and stratified by PRR14 expression is also analyzed (**c**). KM survival curves of breast cancer patients receiving chemotherapy with mutant P53 (**d**) or wild-type P53 (**e**) are stratified by their expression level of PRR14.

Correspondingly, when treated with BML, cell lines show similar effect of PRR14 overexpression (Fig. 7c, d). In wild-type *P53* cell lines, the percentage of Eto-induced 4N cells is reduced, which negatively correlates with BML concentration (MCF7: *P* = 0.030, *R*^2^ = 0.655; 10AKRAS: *P* < 0.001, *R*^2^ = 0.866), while in P53 non-functional cell lines including 7E6 and MDA-MB-231, the percentage of arrested G2 cells is not regulated by CHEK2 inhibitor. As genotoxic reagents including Eto are involved in the chemotherapy of breast cancer, we check the influence of CHEK2 and PRR14 on patients’ response to chemotherapy. In the TCGA database, high *CHEK2* expression is correlated with better relapse-free survival (Fig. 8a, *P* = 0.002). As expected, high *PRR14* expression has an opposite effect and results in worse relapse-free survival curve (Fig. 8b*, P* = 0.003). In contrast, patients’ response to endocrine therapy is hardly influenced by *PRR14* expression (Fig. 8c, *P* = 0.31). In light of the importance of *P53* in cancer and PRR14’s regulation on its activity, we also check the impact of *P53*’s status on PRR14’s effect. Just as we previously hypothesized, the effect of PRR14 is influenced by the status of *P53*. In mutant *P53* patients, there is barely any difference in the survival rate between differential PRR14 expressing patients (Fig. 8d*, P* = 0.33). However, in wild-type *P53* patients, high level of PRR14 is associated with worse relapse-free survival, though the effect is insignificant (Fig. 8e*, P* = 0.061), which might be due to insufficient number of patients.

## Discussion

Previous data analysis demonstrates that *PRR14* transcripts are elevated in various types of cancer^20^. Here we confirm *PRR14* gene is amplified and overexpressed in breast cancer (Figs. 1 and 2). However, gene amplification only contributes to <5% of *PRR14* overexpression, the majority of which is a consequence of upregulated transcription and the underlying mechanism is still unknown. On the other hand, *PRR14* is rarely mutated. Thus, its function in oncogenesis is mainly mediated by overexpression, which implies PRR14 has a rather conservative and important role during oncogenesis.

The PI3K/AKT/mTOR signaling pathway is one of the most frequently dysregulated pathways in cancer^38,39^. PRR14 overexpression and its function in activating the PI3K/AKT/mTOR signaling pathway have already been demonstrated in lung cancer^21^. Here in breast cancer, both differential expression analysis and in vitro biochemistry analysis show PRR14 strongly activates the signaling pathway as well (Figs. 3 and 4). This implies PRR14 overexpression may be a common mechanism contributing to the PI3K/AKT/mTOR signaling pathway dysregulation in various types of cancer. The evidence of PRR14 overexpression activating the PI3K/AKT/mTOR signaling pathway in other types of cancer is needed.

Among all the PRR14-overexpressing cancers, breast cancer shows the most significant increase of PRR14^20^. Therefore, we suspect PRR14’s function in breast cancer may not be limited to the activation of the PI3K/AKT/mTOR signaling pathway. Noticeably, the ATM signal pathway appears in the result of differential expression analysis (Fig. 4c, Supplementary Table 3), which is absent in the analysis of lung cancer^21^. And PRR14 is able to regulate cell cycle in multiple ways: (1) PRR14 protects cells from apoptosis and decrease of PRR14 lowers cells’ proliferation rate (Fig. 5a, b), which is consistent with PRR14’s impact on growth curve; (2) PRR14 overexpression regulates cell cycle G2/M checkpoint (Figs. 5 and 7). Further analysis and experiments pinpoint *CHEK2* as the mediator between PRR14 and the ATM signal pathway (Figs. 6 and 7): (1) Inhibiting CHEK2 activity perfectly mimics the effects of PRR14 overexpression (Fig. 7). (2) Additional PRR14 decreases the transcription of *CHEK2*, resulting in its downregulation (Fig. 6). (3) The activation of CHEK2 in response to genotoxicity is partially inhibited by PRR14 (Fig. 6). (4) Although PRR14 shows almost no impact on endocrine therapy (Fig. 8e), CHEK2 and PRR14 have opposite impacts on breast cancer patients receiving chemotherapy (Fig. 8a–c). (5) The status of P53, main effector of CHEK2, greatly influences PRR14’s impact on cell cycle in response to DNA damage (Figs. 6–8). Altogether, we conclude PRR14 regulates cell cycle through CHEK2. As tumor suppressor *CHEK2* is widely accepted as a driver gene during tumorigenesis in various types of cancer, mainly in breast cancer^32,33^, PRR14’s inhibition of *CHEK2* may explain its more powerful oncogene function in breast cancer and predict poor response to chemotherapy in PRR14-overexpressing patients.

In summary, our results suggest for the first time, that *PRR14* promotes breast tumorigenesis mainly through overexpression, which further activates the PI3K/AKT/mTOR pathway and inhibits the CHEK2 pathway. The importance of the two pathways in breast cancer is well established. PRR14 here provides a novel mechanism mediating their interaction and renders patients more resistance to chemotherapy. Therefore, it may be potential target for therapeutic strategies in breast cancer.

## Materials and methods

### Cell culture, plasmids, transfection and stable cell line establishment

MCF7, MDA-MB-231 and 10AKRAS cells were maintained using standard conditions. MCF7 and MDA-MB-231 were obtained from the Cell Bank of the Chinese Academy of Sciences (Shanghai, China) and validated by STR DNA analysis. Chemicals including selective CHEK2 inhibitor BML-277 (BML, HY-13946, MCE)^46^, and genotoxic chemicals, including bleomycin (Bleo, HY-17565, MCE), etoposide (Eto, HY-13629, MCE), 5-fluorouracil (5-FU, HY-90006, MCE), H_2_O_2_ (88597, Millipore) and hydroxyurea (HU, HY-B0313, MCE), were used to treat cells.

Complementary DNA encoding full-length human PRR14 was cloned into the pQCXIH vector (Clontech) as previously described^19^. DNA transfection was carried out using Lipofectamine 2000 (Invitrogen, Life Technologies, Carlsbad, CA, USA). siRNA (SASI_Hs01_00196781, SASI_Hs01_00196782 and SASI_Hs01_00196783, Sigma, St Louis, MO, USA) transfection was mediated by Lipofectamine RNAiMAX Transfection Reagent (Invitrogen, Life Technologies). Retroviral production and infection were performed following the protocol from Clontech. HEK293T cells were used as packaging cells. After infection, cells were allowed to recover for 24 h before selection with hygromycin for 1 week. Cells transduced with the empty vector were also established and used as control cell lines.

shRNAs corresponding to siRNA sequences above were designed and inserted into pLKO.1 puro. Lentivirus production and infection were performed following the protocol from Addgene. The pool of three target-specific lentiviral particles was used for infection. After 24 h recovery, cells were treated with puromycin for 1 week.

### RNA isolation, reverse transcription and qPCR

Total RNA was harvested from cells or tissues using the standard TRIzol method (Life Technologies) and used for the cDNA synthesis with iScript cDNA Synthesis Kit (Bio-Rad, Hercules, CA, USA). Real-time PCR was performed with SYBR Green JumpStart Taq ReadyMix (Sigma) using the StepOnePlus Real-Time PCR System (Bio-Rad) according to the manufacturer’s protocol. All reactions were run in duplicate. After vortexing, 10 μl aliquots of the mixture were pipetted into each well of a 96-well thin-wall PCR plate (Bio-Rad). PCRs consisted of a denaturing cycle at 94 °C for 2 min, followed by 40 cycles of 15 s at 94 °C and 1 min at 60 °C. Relative mRNA amounts of target genes were calculated after normalization to an endogenous reference gene (18 s unless otherwise stated) and relative to the negative control with the arithmetic formula 2^−ΔΔCt^. The following primer sequences were used for qPCR: PRR14-F: AGTTGAAGATCGCCATCTCAGA; PRR14-R: GCTGGGGTATTGTGGTCCTG; 18s-F: GTAACCCGTTGAACCCCATT; 18s-R: CCATCCAATCGGTAGTAGCG; ACTB-F: CATGTACGTTGCTATCCAGGC; ACTB-R: CTCCTTAATGTCACGCACGAT; CHEK2-F: TTATCTGCCTTAGTGGGTATCCA; CHEK2-R: CTGTCGTAAAACGTGCCTTTG.

### Proliferation assay and fluorescent-activated cell sorting (FACS) analysis

Using alamarBlue™ Cell Viability Reagent (DAL1100, Invitrogen) in 96-well plate at indicated time points and measured by Varioskan Flash multimode reader (Thermo Fisher Scientific), cell proliferation was monitored. Three technical replicates were performed per assay.

For FACS analysis, 80% confluent cells in Corning p35 tissue culture dishes (Corning, NY) were trypsinized, harvested by centrifugation (500 × *g*, 5 min), washed in 4 °C phosphate-buffered saline (PBS) once and fixed in 70% ethanol (in PBS) overnight. Then cells were washed once in 4 °C PBS and stained with propidium iodide (PI) (P4170, Sigma) w/o phospho-Histone H3 (p-H3, #3465, Cell Signaling). Cell-cycle profiles were obtained on a FACSCalibur flow cytometer (Becton Dickenson). Data were analyzed using the FlowJo v10.1 (San Diego, CA).

### Tissue samples, immunohistochemistry and western analysis

Seven cases of breast cancer tissues and paired paracancerous tissues were collected from The Third Affiliated Hospital of Sun Yat-sen University and diagnosed by the Department of Pathology. All patients were informed and agreed. Study protocols of human samples were approved by the Ethics Board of The Third Affiliated Hospital of Sun Yat-sen University and were performed in accordance with all relevant principles of the Declaration of Helsinki.

The human breast cancer tissue microarray (No. BR1921b, Alenobio) was employed to further validate the result from above-mentioned patients, which comprises 80 invasive ductal carcinomas, 80 invasive lobular carcinomas, 29 specimens from cancer adjacent normal breast tissue and 3 specimens from normal breast tissue. Immunohistochemistry was performed according to standard procedure and section was processed with hematoxylin and eosin reagents and stained for PRR14 (Novus, NBP2–31812). The results were semi-quantitative evaluated by 2 pathologists independently, with differences resolved through careful discussion.

For western blot, cells and liquid frozen tissues were lysed in NP-40 buffer supplemented with phosphatase and protease inhibitors (1 mM Na3VO4, 1 mM NaF, 1 mM PMSF, 1 mM EDTA, 1 μg ml^−1^ aprotinin, 1 μg ml^−1^ leupeptin). Western analysis was performed using standard procedures and the protein of interest was normalized to the β-ACTIN protein level. The following antibodies were used: PRR14 (Novus, NBP2–31812), phospho-PDK1(#3438, Cell Signaling), phospho-AKT (#13038, Cell Signaling), phospho-S6K (#9234, Cell Signaling), phospho-S6(#4858, Cell Signaling), β-ACTIN (sc-47778; Santa Cruz), CHEK2 (#2662, Cell Signaling), phospho-CHEK2 (#2197, Cell Signaling), P53 (#18032, Cell Signaling), phospho-P53 (#9287, Cell Signaling), P21 (#2947, Cell Signaling),γ-H2AX (#9718, Cell Signaling) and Flag (clone M5, Sigma).

### Tumor xenografts

MCF7 (5 × 10^6^ suspended in 100 μl Matrigel matrix (354262, BD Biosciences)) and MDA-MB-231 (2.5 × 10^6^ suspended in 100 μl sterile PBS) stable cell lines were subcutaneously injected into dorsal flanks of female nude mice (BALB/c-nu, Hunan SJA Laboratory Animal Co., Ltd, five mice per group) at 6 weeks of age respectively. Mice injected with MCF7 cell lines were fed with estrogen to help tumor formation. Administration of 17β-estradiol (2758, Sigma, E2, 0.10 g/l) in the drinking water was started 1 week before the cell implantation. Palpable tumors were established in 15–30 days after injection. Mice were sacrificed after 6 weeks by CO_2_ inhalation at the experiment endpoint and tumors were dissected and weighed. All animal procedures were conducted in accordance with the Guidelines for the Care and Use of Laboratory Animals and were approved by the Second Xiangya Hospital, Central South University.

### Statistical analysis

Statistical analyses were performed using the GraphPad software package (Prism6, GraphPad Software, La Jolla, CA, USA) and the criterion for significance was set at *P* < 0.05. If not stated, the experiments were repeated at least three times and the mean of the data were indicated. Two-tailed Student’s *t*-test was used to determine the significance of the difference between two groups. And comparisons among more than two groups were made using one-way ANOVA analysis.

### Bioinformatic analysis and cohorts

The gene copy number information and the PRR14 expression information in cancer and normal control were generated with UCSC Xena Browser (https://xenabrowser.net/heatmap/) using:

- GDC TCGA Breast Cancer (BRCA) gene copy number variation (1247 cases).
- TCGA breast invasive carcinoma (BRCA) gene expression by RNAseq (IlluminaHiseq, 1215 cases).
- TCGA breast invasive carcinoma (BRCA) gene expression (AgilentG4502A_07_3 array, 597 cases).

TCGA BRCA data (TCGA, Provisional, 963 cases) including genetic alterations, gene expression, protein expression and molecular subtypes were extracted using the cBioPortal for Cancer Genomics (http://www.cbioportal.org/). The differential expression analysis within subtypes was performed using Gitools (http://gitools.org/home)^47^. The molecular concept-based analysis of over-represented pathways was performed with ConsensusPathDB^48^, defining the significance level at 0.001 and minimum of four genes represented in the pathway.

Relapse-free survival was performed using Kaplan–Meier plotter. The expression level cutoff was auto-selected for PRR14 (218714_at probe) and CHEK2 (210416_at probe).

## Supplementary information

Supplementary figure

Supplementary information

Supplementary table 1

Supplementary table 2

Supplementary table 3

Supplementary table 4

## References

[1] Zink D, Fischer AH, Nickerson JA (2004). Nuclear structure in cancer cells. Nat. Rev. Cancer.

[2] Gradishar WJ (2015). Breast cancer version 2.2015. J. Natl Compr. Cancer Netw..

[3] Elston CW, Ellis IO (1991). Pathological prognostic factors in breast cancer. I. The value of histological grade in breast cancer: experience from a large study with long-term follow-up. Histopathology.

[4] Volpi A (2004). Prognostic relevance of histological grade and its components in node-negative breast cancer patients. Mod. Pathol..

[5] Beck AH (2011). Systematic analysis of breast cancer morphology uncovers stromal features associated with survival. Sci. Transl. Med..

[6] Burke B, Stewart CL (2013). The nuclear lamins: flexibility in function. Nat. Rev. Mol. Cell Biol..

[7] Chow KH, Factor RE, Ullman KS (2012). The nuclear envelope environment and its cancer connections. Nat. Rev. Cancer.

[8] de Las Heras JI, Batrakou DG, Schirmer EC (2013). Cancer biology and the nuclear envelope: a convoluted relationship. Semin Cancer Biol..

[9] Lammerding J (2004). Lamin A/C deficiency causes defective nuclear mechanics and mechanotransduction. J. Clin. Invest.

[10] Ognibene A (1999). Nuclear changes in a case of X-linked Emery-Dreifuss muscular dystrophy. Muscle Nerve.

[11] Chen CY (2012). Accumulation of the inner nuclear envelope protein Sun1 is pathogenic in progeric and dystrophic laminopathies. Cell.

[12] Kayman-Kurekci G (2014). Mutation in TOR1AIP1 encoding LAP1B in a form of muscular dystrophy: a novel gene related to nuclear envelopathies. Neuromuscul. Disord..

[13] Zhang Q (2007). Nesprin-1 and -2 are involved in the pathogenesis of Emery Dreifuss muscular dystrophy and are critical for nuclear envelope integrity. Hum. Mol. Genet..

[14] Sakuma S, D’Angelo MA (2017). The roles of the nuclear pore complex in cellular dysfunction, aging and disease. Semin. Cell Dev. Biol..

[15] Aljada A (2016). Altered Lamin A/C splice variant expression as a possible diagnostic marker in breast cancer. Cell Oncol..

[16] Willis ND (2008). Lamin A/C is a risk biomarker in colorectal cancer. PLoS ONE.

[17] Sun S, Xu MZ, Poon RT, Day PJ, Luk JM (2010). Circulating Lamin B1 (LMNB1) biomarker detects early stages of liver cancer in patients. J. Proteome Res..

[18] Poleshko A (2013). The human protein PRR14 tethers heterochromatin to the nuclear lamina during interphase and mitotic exit. Cell Rep..

[19] Yang M, Yuan ZM (2015). A novel role of PRR14 in the regulation of skeletal myogenesis. Cell Death Dis..

[20] Xiao S, Yang M (2016). Discovery of a novel target for cancer: PRR14. Cell Death Dis..

[21] Yang M, Lewinska M, Fan X, Zhu J, Yuan ZM (2016). PRR14 is a novel activator of the PI3K pathway promoting lung carcinogenesis. Oncogene.

[22] Petsalaki E, Zachos G (2014). Chk2 prevents mitotic exit when the majority of kinetochores are unattached. J. Cell Biol..

[23] Ahn JY, Schwarz JK, Piwnica-Worms H, Canman CE (2000). Threonine 68 phosphorylation by ataxia telangiectasia mutated is required for efficient activation of Chk2 in response to ionizing radiation. Cancer Res..

[24] Matsuoka S (2000). Ataxia telangiectasia-mutated phosphorylates Chk2 in vivo and in vitro. Proc. Natl Acad. Sci. USA.

[25] Shieh SY, Taya Y, Prives C (1999). DNA damage-inducible phosphorylation of p53 at N-terminal sites including a novel site, Ser20, requires tetramerization. EMBO J..

[26] Lee JS, Collins KM, Brown AL, Lee CH, Chung JH (2000). hCds1-mediated phosphorylation of BRCA1 regulates the DNA damage response. Nature.

[27] Bahassi EM (2008). The checkpoint kinases Chk1 and Chk2 regulate the functional associations between hBRCA2 and Rad51 in response to DNA damage. Oncogene.

[28] Yang S, Kuo C, Bisi JE, Kim MK (2002). PML-dependent apoptosis after DNA damage is regulated by the checkpoint kinase hCds1/Chk2. Nat. Cell Biol..

[29] Stevens C, Smith L, La Thangue NB (2003). Chk2 activates E2F-1 in response to DNA damage. Nat. Cell Biol..

[30] Falck J, Mailand N, Syljuasen RG, Bartek J, Lukas J (2001). The ATM-Chk2-Cdc25A checkpoint pathway guards against radioresistant DNA synthesis. Nature.

[31] Ahn J, Prives C (2002). Checkpoint kinase 2 (Chk2) monomers or dimers phosphorylate Cdc25C after DNA damage regardless of threonine 68 phosphorylation. J. Biol. Chem..

[32] Bailey MH (2018). Comprehensive characterization of cancer driver genes and mutations. Cell.

[33] Cancer Genome Atlas Network. (2012). Comprehensive molecular portraits of human breast tumours. Nature.

[34] Pinkel D (1998). High resolution analysis of DNA copy number variation using comparative genomic hybridization to microarrays. Nat. Genet..

[35] Perou CM (2000). Molecular portraits of human breast tumours. Nature.

[36] Sorlie T (2001). Gene expression patterns of breast carcinomas distinguish tumor subclasses with clinical implications. Proc. Natl Acad. Sci. USA.

[37] Parker JS (2009). Supervised risk predictor of breast cancer based on intrinsic subtypes. J. Clin. Oncol..

[38] Shaw RJ, Cantley LC (2006). Ras, PI(3)K and mTOR signalling controls tumour cell growth. Nature.

[39] Engelman JA (2009). Targeting PI3K signalling in cancer: opportunities, challenges and limitations. Nat. Rev. Cancer.

[40] Fischer M, Quaas M, Steiner L, Engeland K (2016). The p53-p21-DREAM-CDE/CHR pathway regulates G2/M cell cycle genes. Nucleic Acids Res..

[41] Reinhardt HC, Aslanian AS, Lees JA, Yaffe MB (2007). p53-deficient cells rely on ATM- and ATR-mediated checkpoint signaling through the p38MAPK/MK2 pathway for survival after DNA damage. Cancer Cell.

[42] Scheffner M, Werness BA, Huibregtse JM, Levine AJ, Howley PM (1990). The E6 oncoprotein encoded by human papillomavirus types 16 and 18 promotes the degradation of p53. Cell.

[43] Martinez-Zapien D (2016). Structure of the E6/E6AP/p53 complex required for HPV-mediated degradation of p53. Nature.

[44] Casamayor A, Morrice NA, Alessi DR (1999). Phosphorylation of Ser-241 is essential for the activity of 3-phosphoinositide-dependent protein kinase-1: identification of five sites of phosphorylation in vivo. Biochem J..

[45] Burma S, Chen BP, Murphy M, Kurimasa A, Chen DJ (2001). ATM phosphorylates histone H2AX in response to DNA double-strand breaks. J. Biol. Chem..

[46] Arienti KL (2005). Checkpoint kinase inhibitors: SAR and radioprotective properties of a series of 2-arylbenzimidazoles. J. Med. Chem..

[47] Perez-Llamas C, Lopez-Bigas N (2011). Gitools: analysis and visualisation of genomic data using interactive heat-maps. PLoS ONE.

[48] Kamburov A, Stelzl U, Lehrach H, Herwig R (2013). The ConsensusPathDB interaction database: 2013 update. Nucleic Acids Res..

[49] Ricoult SJ, Yecies JL, Ben-Sahra I, Manning BD (2016). Oncogenic PI3K and K-Ras stimulate de novo lipid synthesis through mTORC1 and SREBP. Oncogene.

