# Supplementary figures and images for: Oncogene PRR14 promotes breast cancer through activation of PI3K signal pathway and inhibition of CHEK2 pathway
Source: Cell Death Dis. 2020 Jun 15;11(6):464. doi: 10.1038/s41419-020-2640-8 (PMC7296039; doi:10.1038/s41419-020-2640-8)

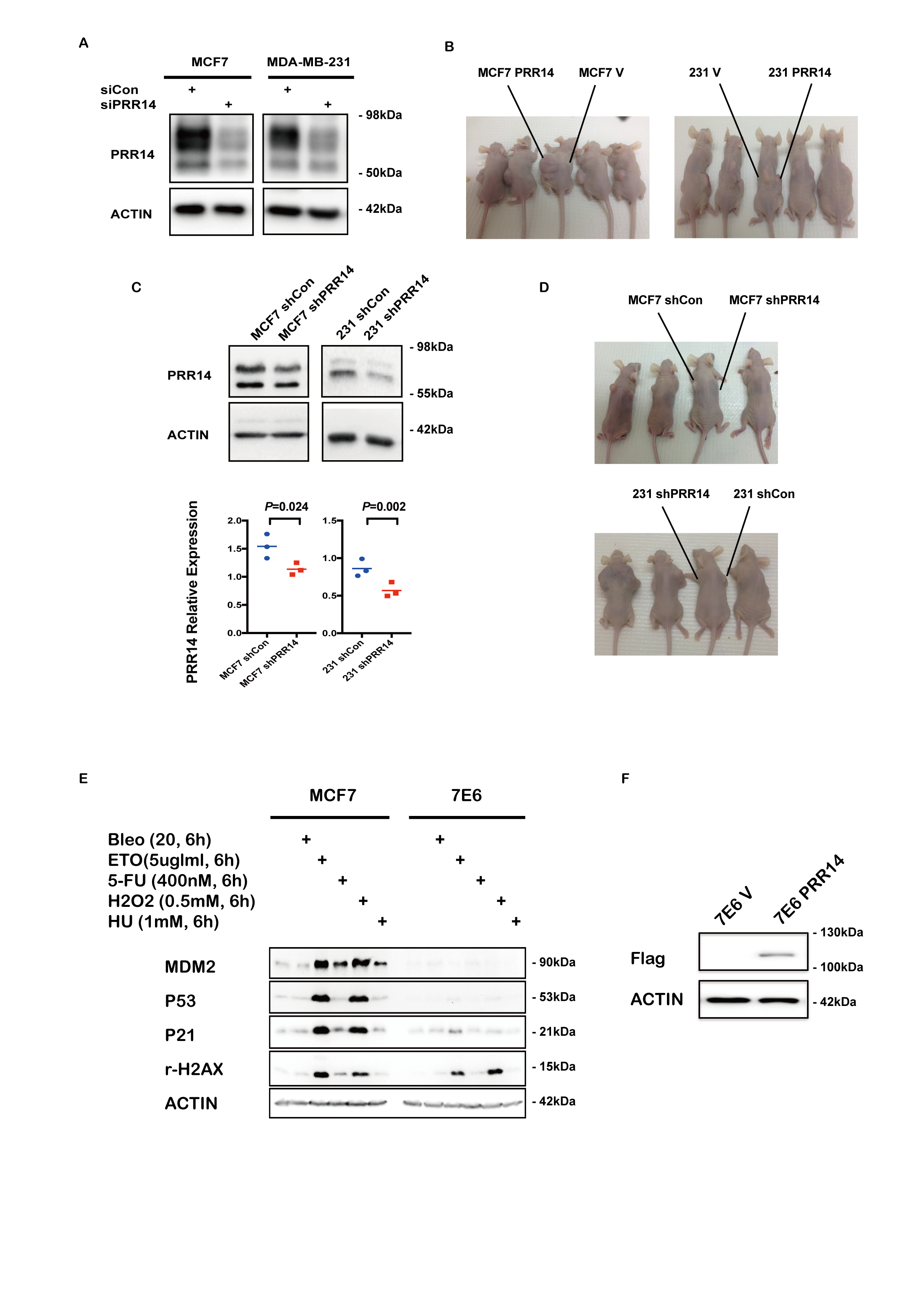

Supplement: Supplementary file 1 — Supplementary figure [file 41419_2020_2640_MOESM1_ESM.png]
