# Supplementary material for: Oncogene PRR14 promotes breast cancer through activation of PI3K signal pathway and inhibition of CHEK2 pathway
Source: Cell Death Dis. 2020 Jun 15;11(6):464. doi: 10.1038/s41419-020-2640-8 (PMC7296039; doi:10.1038/s41419-020-2640-8)
Supplement: Supplementary file 2 — Supplementary information [file 41419_2020_2640_MOESM2_ESM.docx]

**Supplementary information**

1. **Supplementary Materials and Methods**

7E6 cell line was established from MCF7 cells stably transfected with pSG5-E6 [1]. And its PRR14 overexpressing cell line, 7E6 PRR14, as well as the control cell line, 7E6 V, were established by retrovirus as described.

1. **Supplementary files:**

**Supplementary table 1.xls:** **Detailed information of samples in breast cancer tissue microarray and IHC results with anti-PRR14 primary antibody.**

**Supplementary table 2.xls:** **Differentially expressed genes between High- and Low- PRR14 cases in Basal, LumA and LumB subtypes. Gene expression was analyzed by one-way ANOVA analysis and the common 273 DE genes were listed.**

**Supplementary table 3.xls: Significantly enriched pathways based on 273 DE genes**

**Supplementary table 4.xls:** **Differentially expressed proteins between genetically altered and genetically unaltered samples.**

**Supplementary figure.png:** PRR14 depletion by RNAi is verified by immunostaining in both MCF7 and MDA-MB-231 cells (A). Images of tumors form in either side of mice injected with MCF7 PRR14 and its control MCF7 V cells, or 231 PRR14 and its control 231 V cells (B). (C) PRR14 protein level is detected by immunostaining and quantified with paired Two-tailed Student's t-test in lentivirus-mediated PRR14 depleting cells, which are employed for tumor xenograft in mice. Images of formed tumor are shown (D). (E) Establishment of 7E6 cell line. P53 in 7E6 cell line is degraded by HPV16 E6. 7E6 and its parent cell line MCF7 are treated with various genotoxic chemicals including Bleo, Eto, 5-FU, H_2_O_2_ and HU. γ-H2AX, P53 and its downstream effector MDM2 and P21 are blotted. (F) Establishment of 7E6 PRR14 and its control cell lines. PRR14 overexpression is verified in 7E6 PRR14 cell line by Flag tag.

**3. Additional references:**

1. Gu J, Rubin RM, Yuan ZM (2001) A sequence element of p53 that determines its susceptibility to viral oncoprotein-targeted degradation. Oncogene 20 (27):3519-3527. doi:10.1038/sj.onc.1204454
